# Immunoproteasome components LMP2, PSME1, and PSME2 as novel tissue biomarkers predicting response and survival in neoadjuvant chemoimmunotherapy for resectable NSCLC

**DOI:** 10.3389/fimmu.2025.1654573

**Published:** 2025-09-16

**Authors:** Ru Xie, Ke Zhai, Jinming Yu, Miaoqing Zhao

**Affiliations:** ^1^ Department of Radiation Oncology, Shandong Cancer Hospital and Institute, Shandong First Medical University and Shandong Academy of Medical Sciences, Jinan, China; ^2^ Department of Pathology and Lab Medicine, Shandong Cancer Hospital and Institute, Shandong First Medical University and Shandong Academy of Medical Sciences, Jinan, China; ^3^ Department of Pathology, Shandong Cancer Hospital and Institute, Shandong First Medical University and Shandong Academy of Medical Sciences, Jinan, China

**Keywords:** neoadjuvant immunotherapy combined with chemotherapy, predictive markers, NSCLC, LMP2, PSME1, PSME2

## Abstract

**Background:**

While neoadjuvant chemoimmunotherapy (NACI) improves outcomes in resectable non-small cell lung cancer (NSCLC), a significant subset of patients exhibits innate resistance. Biomarkers predicting response are urgently needed. Given the central role of antigen processing in immunotherapy efficacy, we investigated key immunoproteasome components—LMP2 (PSMB9), PSME1, and PSME2—as potential tissue-based biomarkers for NACI response and survival.

**Methods:**

Potential biomarker genes were identified through systematic literature review of NSCLC immunotherapy transcriptomic datasets. Candidate genes underwent validation in public databases (GEO, TCGA) via differential expression and Kaplan-Meier survival analysis. Protein expression of LMP2, PSME1, and PSME2 was assessed by immunohistochemistry (IHC) in pre-treatment tumor biopsies from a retrospective cohort of 50 resectable NSCLC patients treated with NACI (platinum-based chemotherapy + anti-PD-1/PD-L1). Pathologic response was categorized as major pathologic response (MPR, ≤10% residual viable tumor) or incomplete pathologic response (IPR). Associations with MPR, overall survival (OS), and independent prognostic value were evaluated.

**Results:**

Bioinformatic analysis identified LMP2, PSME1, and PSME2 as immunoproteasome subunits linked to antigen presentation pathways. In the clinical cohort, low pre-treatment intratumoral expression of LMP2, PSME1, and PSME2 (by IHC) significantly predicted MPR (*P* < 0.05). Specifically, IPR patients exhibited higher median IHC scores for all three proteins compared to MPR patients. Kaplan-Meier analysis demonstrated that high pre-treatment LMP2 expression was associated with significantly improved OS (median OS: Not Reached vs. 40.0 months, *P <*0.0104). Post-NACI pathological stage (ypTNM III-IV) correlated with worse OS (*P* = 0.0027). Multivariate Cox analysis confirmed MPR status (HR = 8.709, *P* = 0.003), and high pre-treatment LMP2 (HR = 0.051, *P* = 0.007) as independent prognostic factors for OS.

**Conclusion:**

Low pre-treatment expression of immunoproteasome subunits LMP2, PSME1, and PSME2 predicts favorable pathologic response to NACI in resectable NSCLC. High baseline LMP2 expression, along with MPR achievement, independently associates with improved survival. These findings nominate LMP2/PSME1/PSME2 as novel, IHC-detectable biomarkers for stratifying NACI response and prognosis, highlighting the critical role of antigen processing machinery in modulating treatment efficacy. Validation in larger prospective cohorts is warranted.

## 1 Introduction

Lung cancer is the leading cause of cancer‐related deaths worldwide. The American Cancer Society will release cancer statistics data in 2024, showing that lung cancer death rate ranks first, almost 2.5 times that of the second place in cancer death rate. Lung cancer causes far more deaths each year than colorectal, breast, and prostate cancers combined, with over 85% of these cases classified as NSCLC (1), Surgery is the main radical treatment for NSCLC; however, direct surgery is difficult in some patients with stage III NSCLC, especially the N2 stage. Neoadjuvant therapy was delivered to these patients to reduce the tumor stage, improve operability, and eliminate micro‐metastatic disease. Similarly, neoadjuvant therapy can also be used in patients with early and middle stage NSCLC to improve patient prognosis. In recent years, therapeutic strategies harnessing the immune system to eliminate tumor cells have been successfully used for several cancer types, including in patients with advanced-stage NSCLC. In these patients, immune-checkpoint inhibitors (ICIs) can provide durable responses and improve overall survival either as monotherapy, or combined with chemotherapy or other immunotherapeutic agents (2). The advent of immune checkpoint inhibition has pushed the treatment paradigm for resectable NSCLC toward neoadjuvant therapy. A growing number of promising trials have examined the utility of neoadjuvant immunotherapy, both alone and in combination with other modalities such as radiation therapy (RT) and chemotherapy (3). Recently, several clinical trials have been initiated to evaluate the effectiveness of neoadjuvant immunotherapy in combination with chemotherapy. CheckMate - 816 (4) as a new auxiliary immune MDT first phase III randomized controlled clinical studies, the results show that Neoadjuvant immunotherapy combined with chemotherapy significantly improved the pathological complete response (pathological complete response, PCR) rate (24.0% vs. 2.2%, *P* < 0.01), and median Event-free survival (24.0% vs. 2.2%, *P* < 0.01) EFS) (31.6 months vs. 20.8 months, HR = 0.63, *P* = 0.005) significantly decreased the total lung resection rate of stage III NSCLC and significantly extended the 3-year OS (5). KEYNOTE-671 study (6), NADIM II study (7), AEGEAN study (8), Neotorch study (9), and ongoing IMPOWER030, RATIONALE-315, and SHR-1316-III-303 Et al. related studies (10) all showed that neoadjuvant immunotherapy combined with chemotherapy can significantly improve MPR and EFS of resectable NSCLC compared with neoadjuvant chemotherapy.

Unfortunately, a considerable percentage of NSCLC do not completely respond to therapy, which has been associated with early disease progression (11). This finding indicated the urgency of identifying proper markers to effectively predict patients’ responses to NACI in NSCLC.

In this study, we first searched articles related to NSCLC immunotherapy RNA sequencing or single-cell sequencing, and recorded top15–20 genes with good and poor efficacy respectively to obtain the first dataset. In the second step, the genes of dataset 1 were verified by public database (gene difference analysis, Kaplan-Meier analysis, etc.), and three genes with significant differences were finally screened -LMP2, PSME1, and PSME2. PSME1 and PSME2 encode the IFN-γ-induced PA28 alpha β complex, which binds and enhances the peptide synthesis capacity of constitutive and immune proteasomes. LMP2 (PSMB9) is located in the Class II region of the MHC (Major Histocompatibility Complex). Interferon gamma induces the expression of this gene, and this gene product replaces the catalytic subunit 1 (beta 6 subunit) in the immune proteasome. We performed IHC to examine the expression levels of LMP2, PSME1 and PSME2 in NACI-NSCLC tumor tissue samples before NACI, and to explore their prognostic value in patients with NACI-NSCLC. We found that LMP2, PSME1 and PSME2 were significantly depleted in responders, and the IHC score of LMP2, PSME1 and PSME2 in IPR patients was higher than that in MPR patients. Kaplan-Meier analysis showed that, pathological tumor, lymph node and metastatic stage (cTNM) before NACI, pathological tumor, lymph node and metastatic stage after NACI (ypTNM), low LMP2 before NACI, low PSME1 before NACI, and low PSME2 before NACI are good prognostic factors for patients with NACI-NSCLC. In addition, multivariate Cox analysis showed that MPR and LMP2 before NACI were independent prognostic factors for patients with NACI-NSCLC.

## 2 Materials and methods

### 2.1 Patients

All NSCLC cases surgically resected post‐NACI at Shandong Cancer Hospital and Institute between December 2020 and September 2023 were retrieved. The inclusion criteria were as follows: (1) stage I–III NSCLC; (2) a biopsy for diagnosis and surgery performed in our hospital; and (3) paraffin‐embedded tissue sections reserved in our hospital; (4) A pathologist confirmed the presence of sufficient tumor material in the section. Informed consent was obtained from patients or authorized persons. The exclusion criteria were as follows: (1) small cell lung cancer; (2) R1 or R2 resection; and (3) other treatments before surgery, such as radiotherapy, targeted therapy, anti‐angiogenesis therapy, and immunotherapy. All patients received at least two cycles of NACI and radical surgery. The chemotherapy regimens were combined platinum and pemetrexed, paclitaxel, or gemcitabine. The immunotherapy regimen includes one of the following drugs: sintilimab, toripalimab, tislelizumab, camrelizumab, nabuliumab, or atezolizumab. The surgical approaches included thoracotomy and video‐assisted thoracoscopic surgery. CTNM and ypTNM were restaged according to AJCC Cancer Staging (9th edition).

### 2.2 Gene selection and public database validation methods

#### 2.2.1 Candidate gene selection

As of April 24, 2025, we conducted a systematic search of PubMed, Embase, and Web of Science to collect relevant studies. The search terms included combinations of “lung”, “cancer”, “tumor”, “RNA sequencing”, “immunotherapy”, “neoadjuvant chemoimmunotherapy”, “immune microenvironment”, and “biomarker”. In each study meeting the inclusion criteria, we extracted the top 15–20 genes reported by the original authors as differentially expressed between responders and non-responders. We then counted how many independent studies (or cohorts) listed each gene among their top-ranked candidates. The degree of overlap between gene lists was quantified by intersection counts, and a summary statistics table is provided in **Supplementary Table S1**.

Genes were prioritized for downstream validation if they met any of the following criteria:

1. Appeared in the intersection of multiple independent bulk RNA-seq cohorts (i.e., reported in ≥2 cohorts);
2. Observed consistently in both bulk and single-cell datasets;
3. Classified as part of the antigen processing/presentation pathway in the original study.

To focus on novel tissue biomarkers, we excluded genes that had already been extensively validated at the protein level in previous NSCLC neoadjuvant studies. This screening workflow ultimately yielded a panel of 5–10 candidate genes, which were further validated in public GEO/TCGA datasets and subsequently confirmed in our clinical cohort using IHC.

#### 2.2.2 Survival analysis and differential gene expression

OS and Recurrence-Free Survival (RFS) were visualized and analyzed using the KMPlot website(https://kmplot.com/analysis/) and GEPIA2.0 website (http://gepia2.cancer-pku.cn). Meanwhile, the GEPIA2.0 website (http://gepia2.cancer-pku.cn) can conduct differential expression analysis.

#### 2.2.3 PPI network construction

The LMP2, PSME1, PSME2 protein-protein interaction (PPI) network was constructed using the online network tool Search Tool for the Retrieval of Interacting Genes and Proteins (STRING) (https://www.string-db.org/). DAVID used to analyze the function of enrichment online tools (https://david.ncifcrf.gov/), Annotation of paths and functions is performed based on the KEGG databases (https://www.kegg.jp/) and Gene Ontology (http://geneontology.org/). The screening condition was *P* -value < 0.05 after correction by Benjamini-Hochberg.

### 2.3 MPR, IPR assessment

Pathologic responses were evaluated based on the percentage of residual viable tumor (12). The residual tumor cell rate was independently assessed by at least two senior pathologists, with a third senior pathologist involved in cases with inconsistent results. The residual tumor cells were assessed and recorded from 0% to 100% at 10% intervals, We classified each advanced NSCLC patient into MPR (defined as ≤10% (residual viable tumor cells, RVT) in the resected tumor specimens) (13) or IPR(defined as >10% (residual viable tumor cells, RVT) in the resected tumor specimens).

### 2.4 Immunohistochemical staining

Paraffin‐embedded tumor tissues were cut into 4 μm serial sections and dried at 60°C for 1 h. Tissue sections were deparaffinized in xylene, rehydrated with an alcohol gradient, and washed with purified water. The sections were immersed in EDTA repair solution (pH 9.0), heated at medium heat in microwave to boil, then turned to low heat for 15 min, cooled naturally to room temperature, and washed with PBS buffer for 3 times for 5 min each time. 3% hydrogen peroxide solution was added to the sections and incubated at room temperature for 10 min to block endogenous peroxidase activity. The sections were washed with PBS buffer for 3 times for 5 min each time. TBS solution containing 10% normal serum and 1%BSA was closed at room temperature for 2 hours. Remove the liquid from the tissue (do not rinse) and wipe the area around the section with a paper towel. Sections were incubated with primary anti-LMP2 (Abmart, China, TU389124, dilution 1:200), anti-PSME1 (Abmart, China, TU386098, dilution 1:200) and anti-PSME2 (Abmart, TU721263, dilution 1: 6000) overnight at 4 °C. The next day, remove the sections from the refrigerator and return to room temperature. Wash the sections twice in TBS solution containing 0.025%Triton X-100 and gently agitate them for 5 min each time. Goat anti-rabbit IgG-HRP was incubated at room temperature for 30 min and washed 3 times with PBS for 5 min each time. The section is treated with DAB and the color development time is controlled under the microscope (usually 1–3 min), and the distilled water is terminated. Put the sections into the hematoxylin dye solution for 3–5 minutes, rinse with tap water to return to blue, dehydrate with gradient alcohol, xylene transparent. Finally, the sections were removed from xylene and covered with cover slips.

Clarification: The term ‘pre-treatment’ specifically refers to the expression levels of biomarkers measured in formalin-fixed paraffin-embedded (FFPE) tumor samples obtained via diagnostic biopsy prior to the initiation of neoadjuvant therapy.

### 2.5 Immunostaining scoring analysis

The staining results were semi-quantitatively evaluated by the multiply of staining intensity and the percentage of positive staining cells. The percentage of positive cells was given into four grades: 0 for <25%; 1 for 26%–50%; 2 for 51%–75%; 3 for 51%–75% and 4 for >75%. Staining intensity was assessed by four degrees: 0, negative; 1, weak; 2, moderate; and 3, strong (14). Each section was assayed for ten independent high magnifications (×400) fields to get the average scores. IHC score = Cell staining intensity score x percentage of positive cells score. All sections were independently evaluated by two experienced pathologists in a double-blinded manner. Discrepant cases were reviewed jointly and resolved by consensus with a third senior pathologist.

### 2.6 Statistical analysis

Survival analysis was performed using the Kaplan–Meier method and log rank test; univariate and multivariate Cox regression hazards models were used to evaluate survival risk factors. All factors in the univariate Cox analysis were included in multivariate Cox analysis, and the “Forward LR” method was used for multivariate Cox analysis. H-score cutoffs for LMP2, PSME1, and PSME2 were determined using X-tile software (outcome-based optimization), with overall survival (OS) as the primary endpoint. X-tile examines all possible dichotomizations and selects the cutoff that maximizes the log-rank χ² statistic (i.e., minimizes the log-rank *P*). Then, transformed them into LMP2,PSME1,PSME2 low and high groups (15). All statistical analyses were performed using SPSS 27.0 (IBM, Armonk, NY, USA) and GraphPad Prism 10.0 (GraphPad Software Inc., CA, USA). Statistical significance was set at *P* < 0.05.

## 3 Results

### 3.1 Patient characteristics

**Figure 1:**
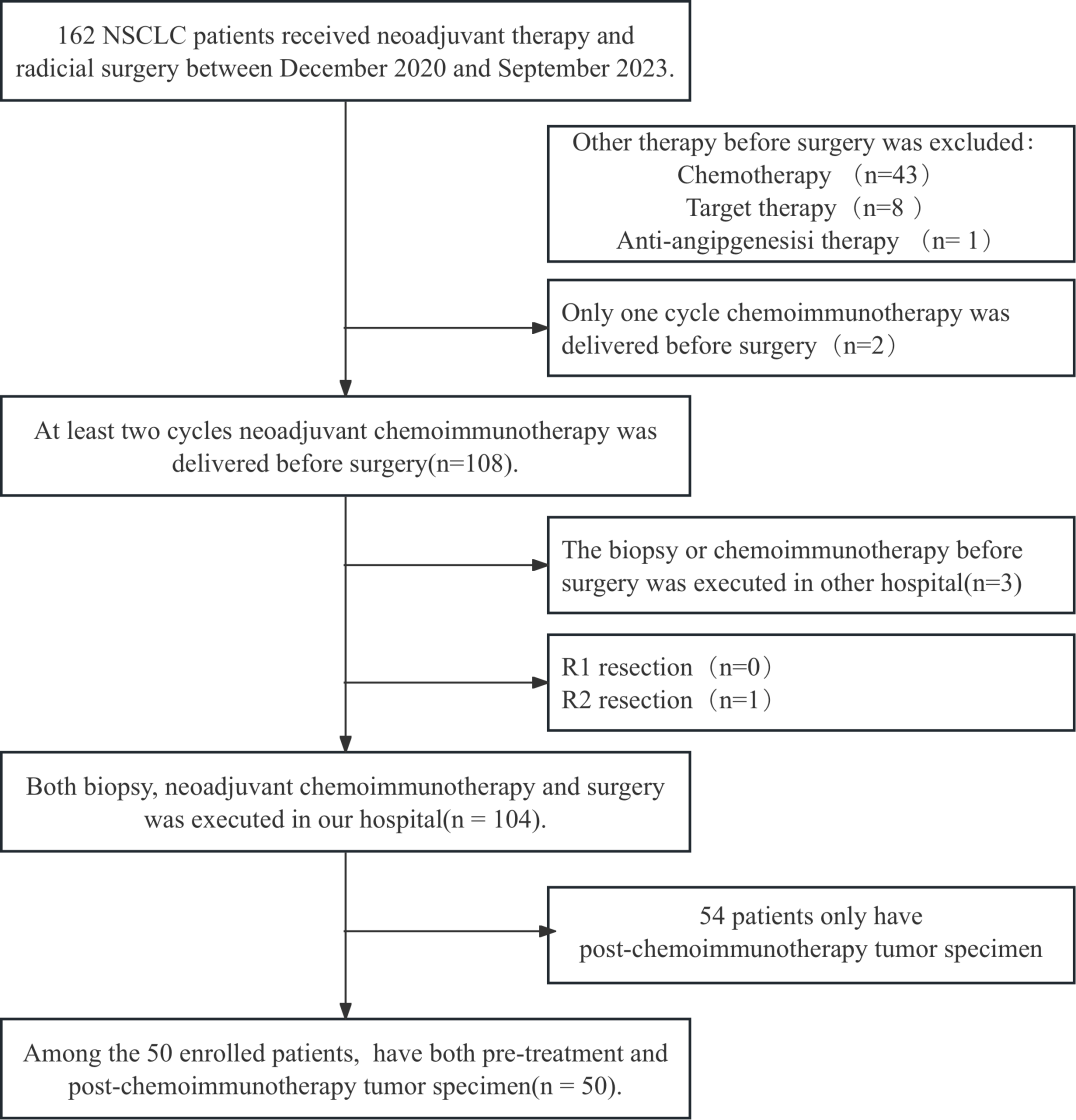
The flowchart for patient recruitment. A total of 162 patients with non-small cell lung cancer (NSCLC) who received neoadjuvant chemoimmunotherapy (NACI) followed by radical surgery in Shandong Cancer Hospital from December 2020 to September 2023 were enrolled. After combining the inclusion and exclusion criteria, 104 patients were enrolled. Fifty of these patients who had paired tumor specimens before and after NACI were included in the final cohort.

All patients with surgically resected NSCLC at Shandong Cancer Hospital and Institute were screened from December 2020 to September 2023 and 50 patients were enrolled according to the inclusion and exclusion criteria. The patient screen flowchart is presented in **Figure 1**. Among them, 86% of the patients were male with a median age of 64 years, and main histopathological types were adenocarcinoma and squamous carcinoma. The median follow‐up time was 24.50months and the median OS was not reached. The distributions of cTNM stages I, II, and III were 2 (4.0%), 10 (20.0%), and 38 (76.0%), respectively. The NACI regimen consists of chemotherapy drugs(platinum combined with pemetrexed, paclitaxel, or gemcitabine)combined with immunodrugs (sindilizumab, tirellizumab, triplizumab, carelizumab, nabuliumab, attilizumab), and all patients received R0 resections. Following NACI, 41 patients experienced descending TNM stages, and 3 patients experienced ascending TNM stages. Among the patients with ascending TNM stages, three had an elevated M stage. The distributions of ypTNM stages I, II, III and IV were 3 (6.0%), 7 (14.0%), 2(4.0%)and 3 (6.0%), respectively. Following NACI, 35 patients achieved MPR; among them, 21 patients achieved a complete pathological response. The clinicopathological characteristics of all patients are presented in **Table 1**.

**Table 1:** Clinicodemographic characteristics of all patients.

Factor	N(%)
Gender(n)	
Male	43(86.0)
Famale	7(14.0)
Age(years), median(range)	67(49-104)
Smoking history(n)	
No	17(34.0)
Yes	33(66.0)
Drinking History(n)	
No	33(66.0)
Yes	17(34.0)
Histopathology	
Adenocarcinoma	9(18.0)
Squamouss	40(80.0)
Adenosquamous carcinoma	1(2.0)
cTNM stage (n)	
I	2(4.0)
II	10(20.0)
III	38(76.0)
ypTNM stage(n)	
MPR	35(70.0)
I	3(6.0)
II	7(14.0)
III	2(4.0)
IV	3(6.0)
Type of ICIs	
Tislelizumab	26(52.0)
Sintilimab	16(32.0)
Camrelizumab	5(10.0)
Navulimab	2(4.0)
Toripalimab	1(2.0)
Pathology response	
MPR	36(72.0)
IPR	14(28.0)
LMP2 H-score	
Low	21(46.7)
High	24(53.3)
PSME1 H-score	
Low	15(37.5)
High	25(62.5)
PSME2 H-score	
Low	32(74.4)
High	11(25.6)
LMP2 H-score,median(range)	4(1-12)
PSME1 H-score,median(range)	4(1-12)
PSME2 H-score,median(range)	6(1-12)

### 3.2 NSCLC ICIs potential biomarker gene retrieval and public data verification

#### 3.2.1 Potential biomarker gene search

We searched articles related to NSCLC immunotherapy RNA sequencing or single-cell sequencing, focusing on the part of differential gene expression, and recorded the end points with good efficacy (CR, PR or PCR, MPR), cohort size, stage, treatment stage, treatment regimen, and the top15–20 genes with good and poor efficacy respectively. Take the intersection and get the first 5–10 genes that can be cross-verified. Proteins that have been reported in relevant articles were highlighted and recorded and removed from the above collection.

In 2020, Thompson et al. used a retrospective cohort study analyzed transcriptional profiles from pre-treatment tumor samples of 51 chemotherapy-refractory advanced NSCLC patients and two independent melanoma cohorts treated with ICB. An antigen processing machinery (APM) score was generated utilizing eight genes associated with APM (*B2M, CALR, NLRC5, PSMB9, PSME1, PSME3, RFX5*, and *HSP90AB1*). Associations were made for therapeutic response, progression-free survival (PFS) and OS. In NSCLC, the APM score was significantly higher in responders compared with non-responders (*P* = 0.0001). An APM score above the median value for the cohort was associated with improved PFS (HR 0.34 (0.18 to 0.64), *P* = 0.001) and OS (HR 0.44 (0.23 to 0.83), *P* = 0.006). It is demonstrated that antigen presentation defect may be an important feature to predict immune checkpoint blockade (ICB) outcome in lung cancer (16).

Hwang S et al. used multipanel markers to predict the response to ICIs by characterizing gene expression signatures or individual genes in patients who showed durable clinical benefit(DCB)to ICIs. Twenty-one patients with NSCLC treated with single-agent anti-programmed cell death protein (PD)-1 antibody were analyzed and their clinicopathological characteristics and response to ICIs were characterized. The results indicate that CD137 and PSMB9 mRNA expression was higher in the DCB group than in the non-durable benefit (NDB) group. Patients with high PSMB9 expression showed longer PFS (17).

By 2023, Ravi et al.to expand our understanding of the molecular features underlying response to checkpoint inhibitors in NSCLC,genomic and transcriptomic analysis of 393 NSCLC patients treated with checkpoint inhibitors identifies molecular features associated with response. Preliminary evaluations identified three genes with prominent functions in lung cancer immunoproteasomes, a nonclassical peptide-processing complex thought to promote differentiation and enhance antigen presentation in the context of proinflammatory cytokines (18), that hold promise as transcriptional predictors: PSME1, PSME2 and PSMB9(LMP2) (19).

According to the above literature retrieval methods, we obtained the first data set, excluded the gene that has been verified by the protein in the literature, and the genes obtained were as follows: CD137, PSME1, PSME2, LMP2, PAK7, VGF, CXCL9, CXCL10, CXCL11, CXCL13, UBD, MMP12, MT1G, PLA2G2D, B2M, CALR, NLRC5, RFX5, HSP90AB1.

#### 3.2.2 Differential gene expression (DEGs) analysis and verification

**Figure 2:**
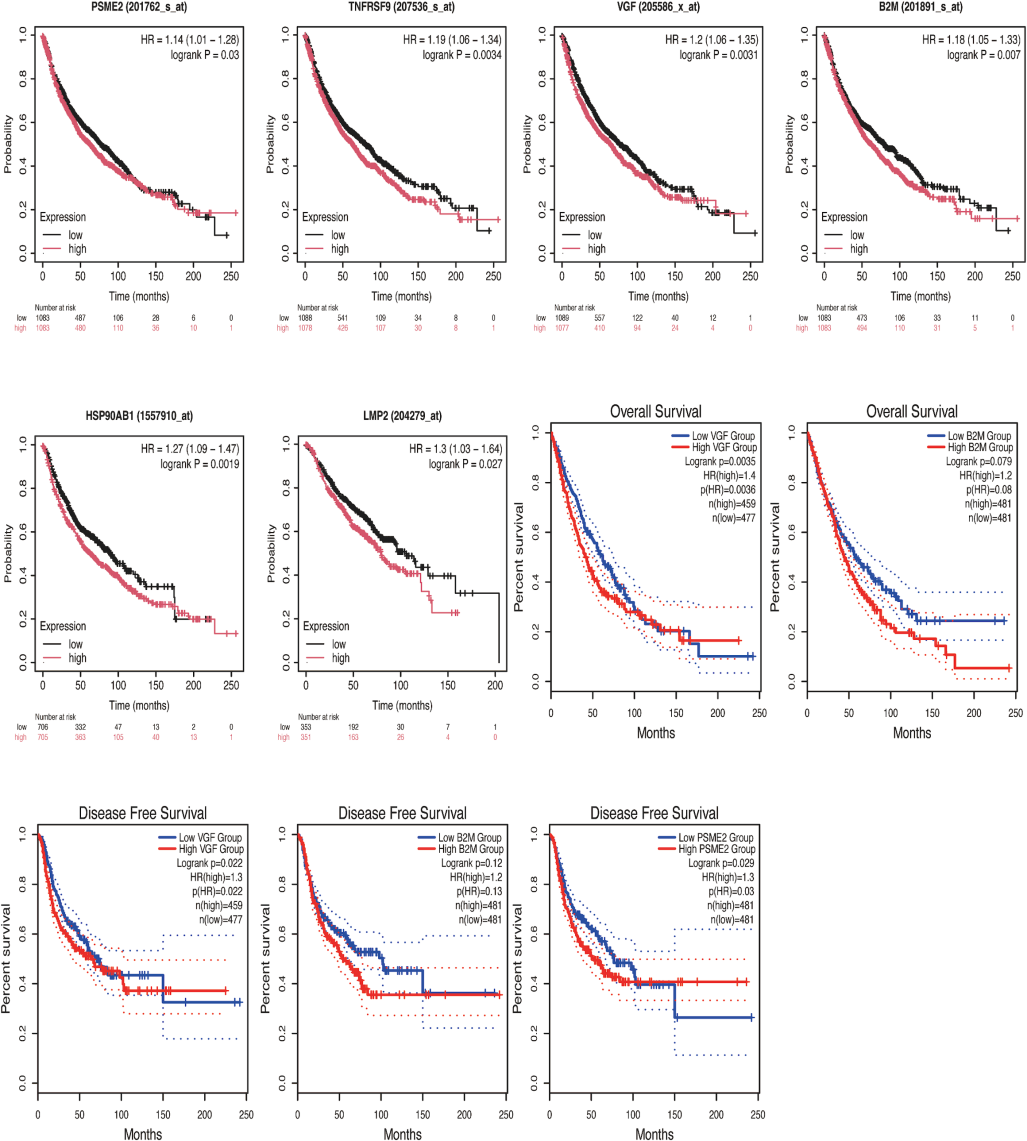
Kaplan-Meier survival curves of high and low expression of PSME2, CD137, VGF, B2M, HSP90AB1 and LMP2 in NSCLC were compared in KMPlot **(A-F)** and GEPIA2.0 **(G-K)**. **(A)** The low-expression group of PSME2(201762_s_at) was associated with better OS (n=2166, *P* = 0.03). **(B)** The low-expression group of CD137[TNFRSF9 (207536_s_at)] was associated with better OS (n=2166,*P* = 0.0034). **(C)** The low-expression group of VGF(205586_x_at) was associated with better OS (n=2166, *P* = 0.0031). **(D)** the low-expression group of B2M(201891_s_at) was associated with better OS (n=2166, P = 0.007). **(E)** The low-expression group of HSP90AB1 (1557910_at) was associated with better OS (n=1411, *P* = 0.0019). **(F)** The low-expression group of LMP2 (204279_at) was associated with better OS (n=704, *P* = 0.027). **(G)** The low-expression group of VGF was associated with better OS (n=936, *P* = 0.0035). **(H)** There was no statistically significant correlation between the B2M low-expression group and the high-expression group and OS [n=962, *P* = 0.079, HR(high)=1.2]. **(I)** The low-expression group of VGF was associated with better DFS (n=936, *P* = 0.022). **(J)** There was no statistically significant correlation between the low-expression group and the high-expression group of B2M and DFS [n=962, *P* = 0.12, HR(high)=1.2]. **(K)** The low-expression group of PSME2 was associated with better DFS (n=962,*P* = 0.029).

We explored the data from TCGA and GEO databases in depth. First, for the GEO database, dataset 1 genes were analyzed using Kaplan-plot tools to assess prognostic significance. The results showed that among 2166 NSCLC patients in the public database, the group with low expression of PSME2, CD137, VGF, B2M and HSP90AB1 was associated with better OS, and the hazard ratio (HR) was greater than 1 (**Figures 2A–E**; *P* < 0.05), and among 704 patients with negative surgical margins, the low LMP2 expression group had a better survival benefit than the high LMP2 expression group, and the HR was greater than 1 (**Figure 2F**; *P* < 0.05). Given the limited availability of data in the GEO dataset, we performed a comprehensive analysis by seamlessly integrating gene expression data from the TCGA database. Comprehensive analysis showed that low VGF and B2M expression were correlated with better OS (**Figures 2G–J**; HR>1, *P* < 0.05), low PSME2 expression had better survival benefits, and HR was greater than 1 (**Figure 2K**; *P* < 0.05).

**Figure 3:**
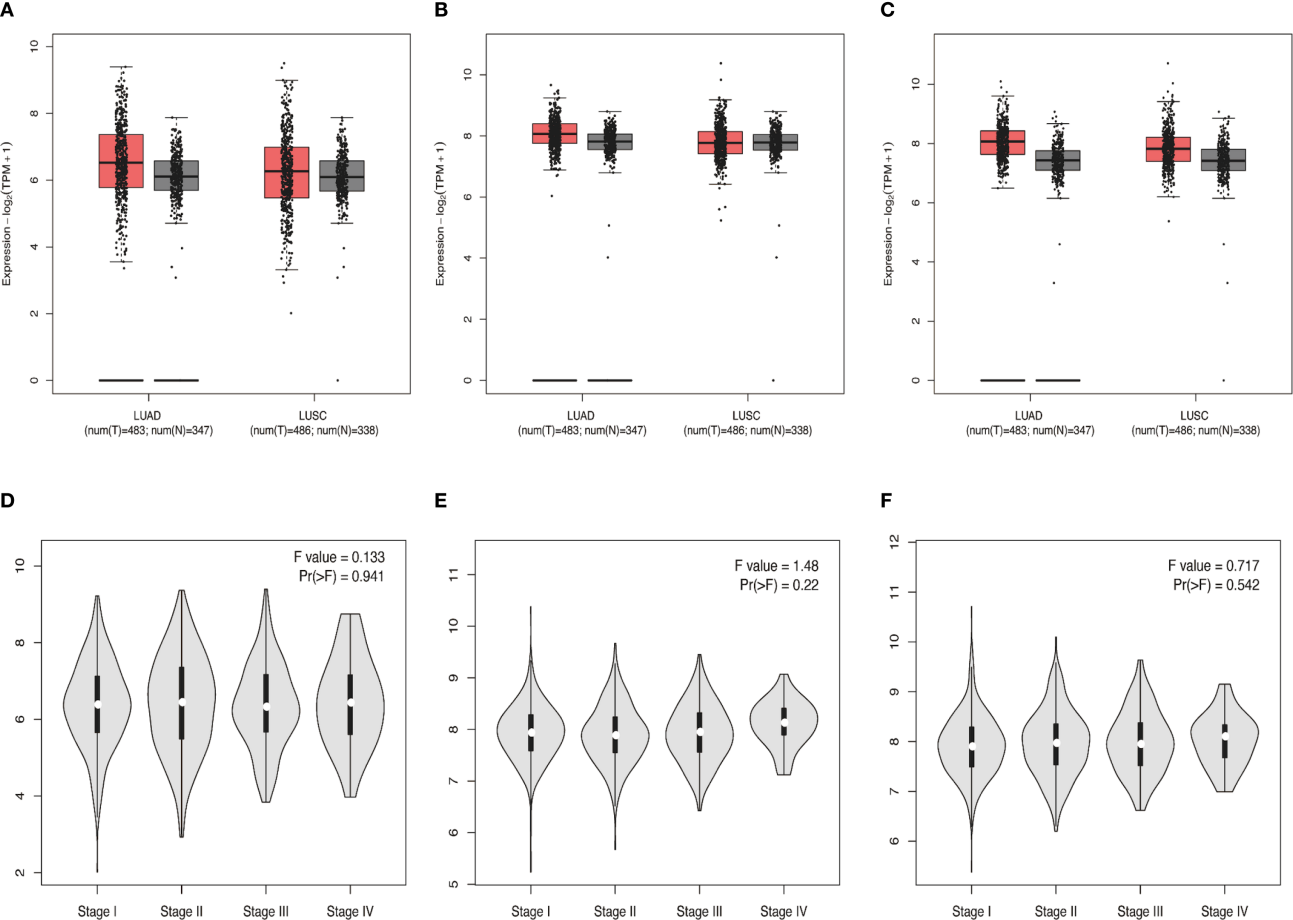
**(A-C)** Differential expressions of LMP2, PSME1, and PSME2 in NSCLC (squamous cell carcinoma, adenocarcinoma) tissues and normal tissues from the TCGA and GETx databases. **(D-F)** The protein expression levels of LMP2, PSME1, and PSME2 at different pathological stages. **(A-C)** The box plot illustrates the expression comparison between lung adenocarcinoma (LUAD) and lung squamous cell carcinoma (LUSC) cancer tissues and normal tissues. Red represents cancer tissues and gray represents normal tissues. The median expression levels of LMP2, PSME1 and PSME2 in lung adenocarcinoma and lung squamous cell carcinoma tissues were higher than those in normal tissues. **(D-F)** The violin diagram indicates that the expression levels of LMP2, PSME1, and PSME2 are similar at different pathological stages of cancer (stages I, II, III, and IV).

Considering that proteins LMP2, PSME1 and PSME2 are all components of proteasome complex and participate in proteasome protein decomposition and immune system biological processes, we decided to select proteins LMP2, PSME1 and PSME2 for NSCLC biomarker exploration. We further analyzed the expression of proteins LMP2, PSME1, and PSME2 in cancer and normal tissues, involving pathological staging. The data showed that the expressions of LMP2, PSME1 and PSME2 in NSCLC tissues were higher than those in normal tissues (**Figures 3A–C**). However, no correlation was observed between protein expression and pathological stage (**Figures 3D–F**). In summary, our preliminary evaluation identified three proteins with prominent functions in the NSCLC proteasome complex and promising biomarkers for NSCLC: LMP2, PSME1, and PSME2.

#### 3.2.3 Biological functions of LMP2, PSME1, and PSME2

**Figure 4:**
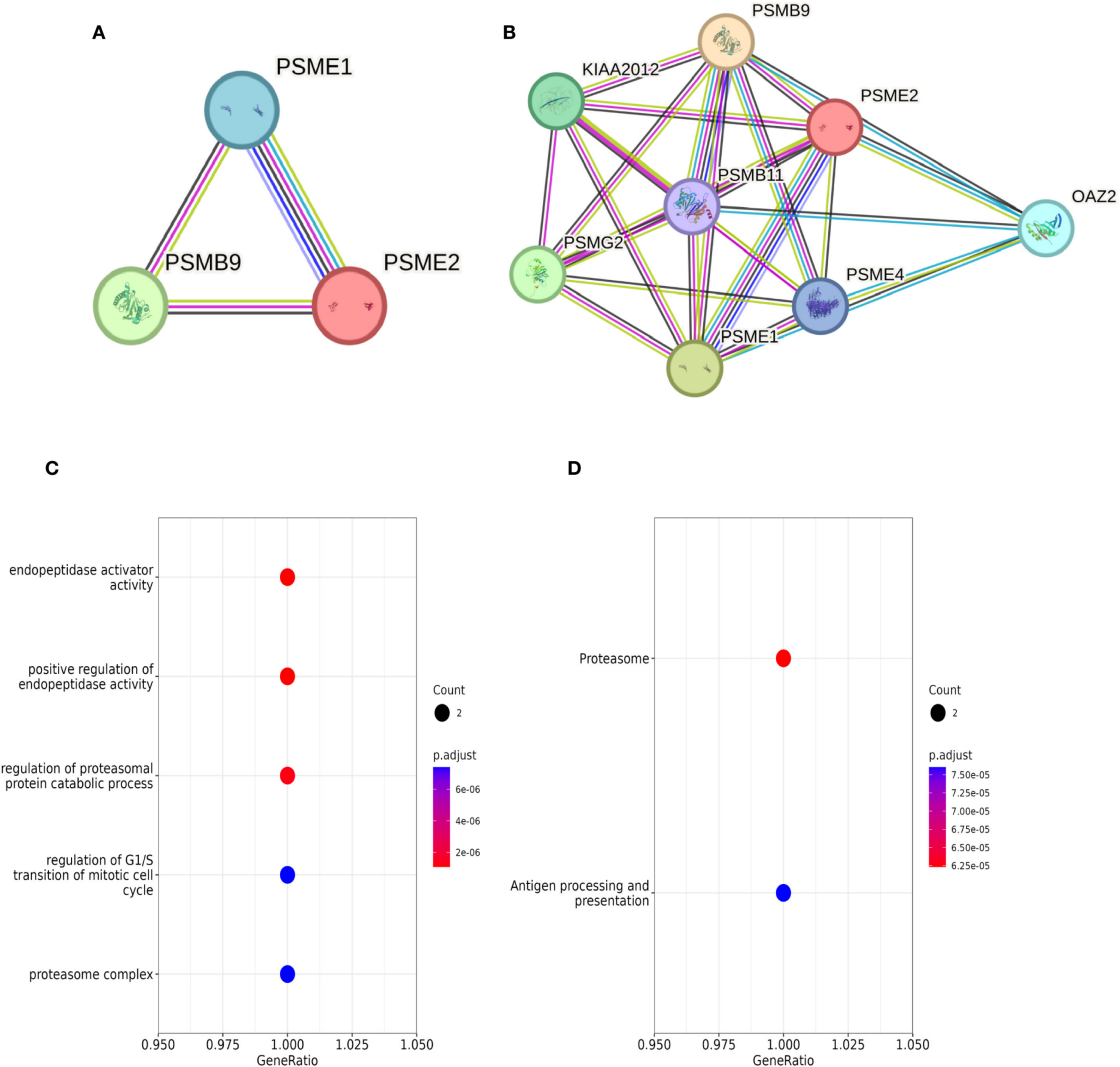
LMP2, PSME1, PSME2 enrichment analysis. **(A, B)** Interaction networks of LMP2, PSME1, and PSME2 proteins retrieved using the protein-Protein Interaction Search tool (STRING). **(C, D)** Functional enrichment analysis of Go and KEGG pathways related to LMP2, PSME1 and PSME2 co-expression genes.

Our research begins by analyzing the interactions between proteins. Subsequently, we performed functional enrichment analysis. In the STRING database, we identified interactions between LMP2, PSME1, and PSME2 proteins, and obtained five proteins that strongly interact with three known proteins: PSMB11, PSMG2, PSME4, OAZ2, and KIAA2012. Together, these proteins form the PPI network (**Figures 4A, B**). In addition, through Go and KEGG functional enrichment analysis, our study revealed the key roles played by LMP2, PSME1 and PSME2 in a variety of biological processes. These processes include proteasome complex composition, antigen processing and presentation, proteasome protein catabolic processes, positive regulation of endopeptase activity, and regulation of mitotic cell cycle G1/S conversion (**Figures 4C, D**). These findings highlight the versatility of LMP2, PSME1, and PSME2 in cell biology and immunology.

### 3.3 Intratumoral LMP2, PSME1 and PSME2 predict responses to NACI in cancer patients

**Figure 5:**
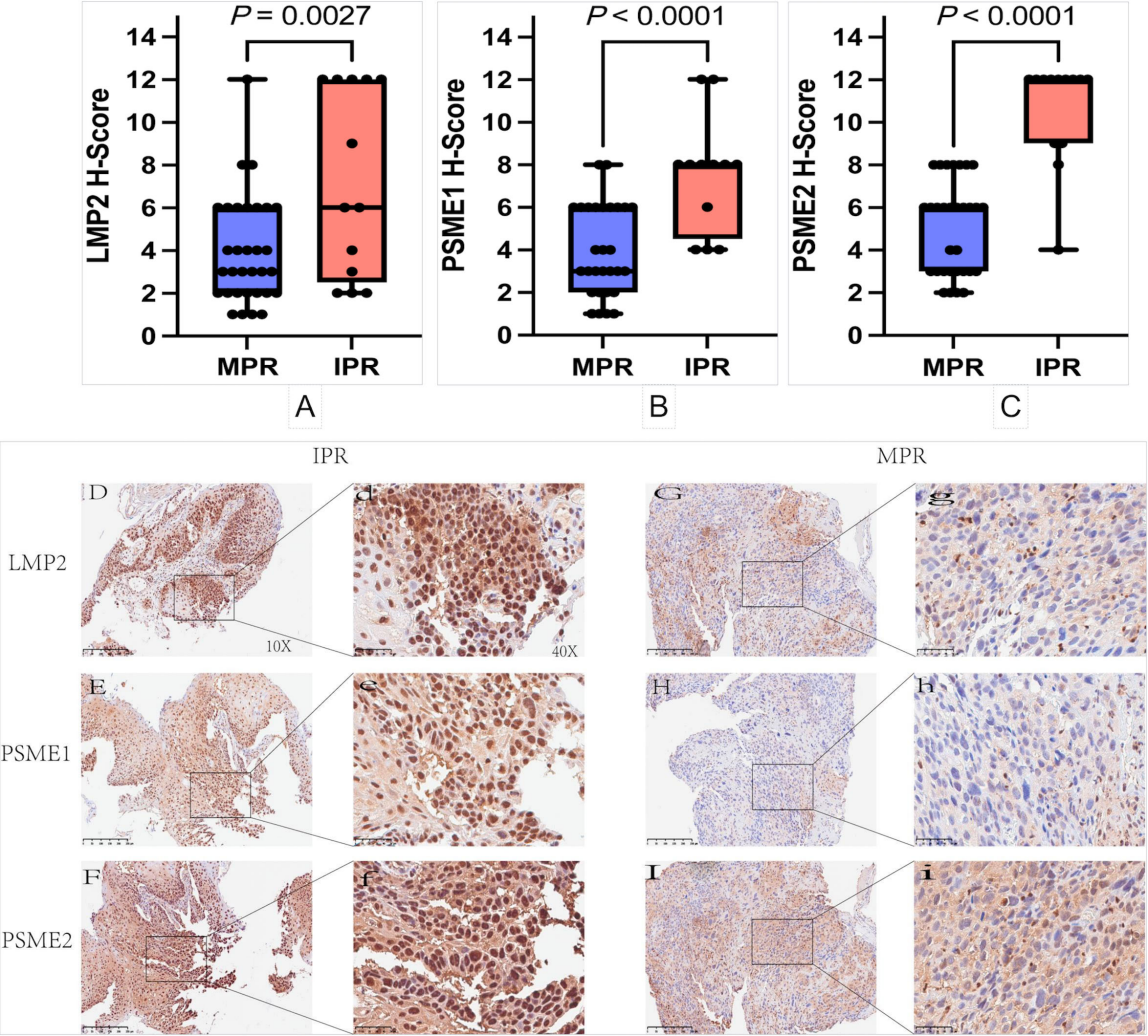
Immunohistochemical staining and analysis. **(A-C)** Relationship between high and low expression of LMP2, PSME1 and PSME2 and pathological response in patients with NSCLC. **(D-I)** The represented immunohistochemistry staining results of two patients who achieved a complete pathological response and non‐major pathology response (MPR) post‐NACI. The represented 10× and 40× images of patient LMP2, PSME1 and PSME2 expressions achieved complete pathological response in naïve tumor **(G**, g-**I**, i**)**. The represented 10× and 40× images of LMP2 expression and PSME1 expression and PSME2 expression of IPR patient in naïve tumor **(D**, d-**F**, f**)**.

Since our analysis above revealed that LMP2, PSME1 and PSME2 might play a role in antitumor immunity, we established a retrospective tumor cohort (validation cohort) of 50 patients with NSCLC treated with NACI to further examine our findings. Tumor samples were collected from the patients before receiving NACI treatment, and the Intratumoral proportion and expression intensity of LMP2, PSME1 and PSME2 was evaluated by performing IHC. We found that LMP2, PSME1 and PSME2 were significantly reduced in responders (**Figures 5A–C**). To better confirm the contribution of LMP2, PSME1 and PSME2 to NACI efficacy, the pathological responses were evaluated based on the percentage of RVT cells (12). Therefore, we classified each of the 50 patients into the group with either MPR or IPR. Since the MPR met the criteria for a surrogate endpoint after neoadjuvant therapy in a variety of cancers, it was strongly associated with improved survival, which is also reflective of the treatment impact and captures the magnitude of the treatment benefit on patient survival (20). As expected, the IHC score of LMP2, PSME1 and PSME2 in IPR patients was higher than that of MPR patient. The LMP2 IHC score for the median MPR and IPR was 3.0 and 6.0, the PSME1 IHC score was 3.0 and 8.0, and the PSME2 IHC score was 6.0 and 12.0. Therefore, we conclude that low intratumoral expression of LMP2, PSME1 and PSME2 predicts a good response to NACI in cancer patients. Representative LMP2, PSME1 and PSME2 immunohistochemical staining results of two patients who had significant increase or decrease following NACI are presented in **Figures 5D–I**. One of them achieved complete pathological response and the other did not achieve MPR.

### 3.4 High pre-treatment LMP2 and ypTNM are favorable prognostic factors for NACI-NSCLC

**Figure 6:**
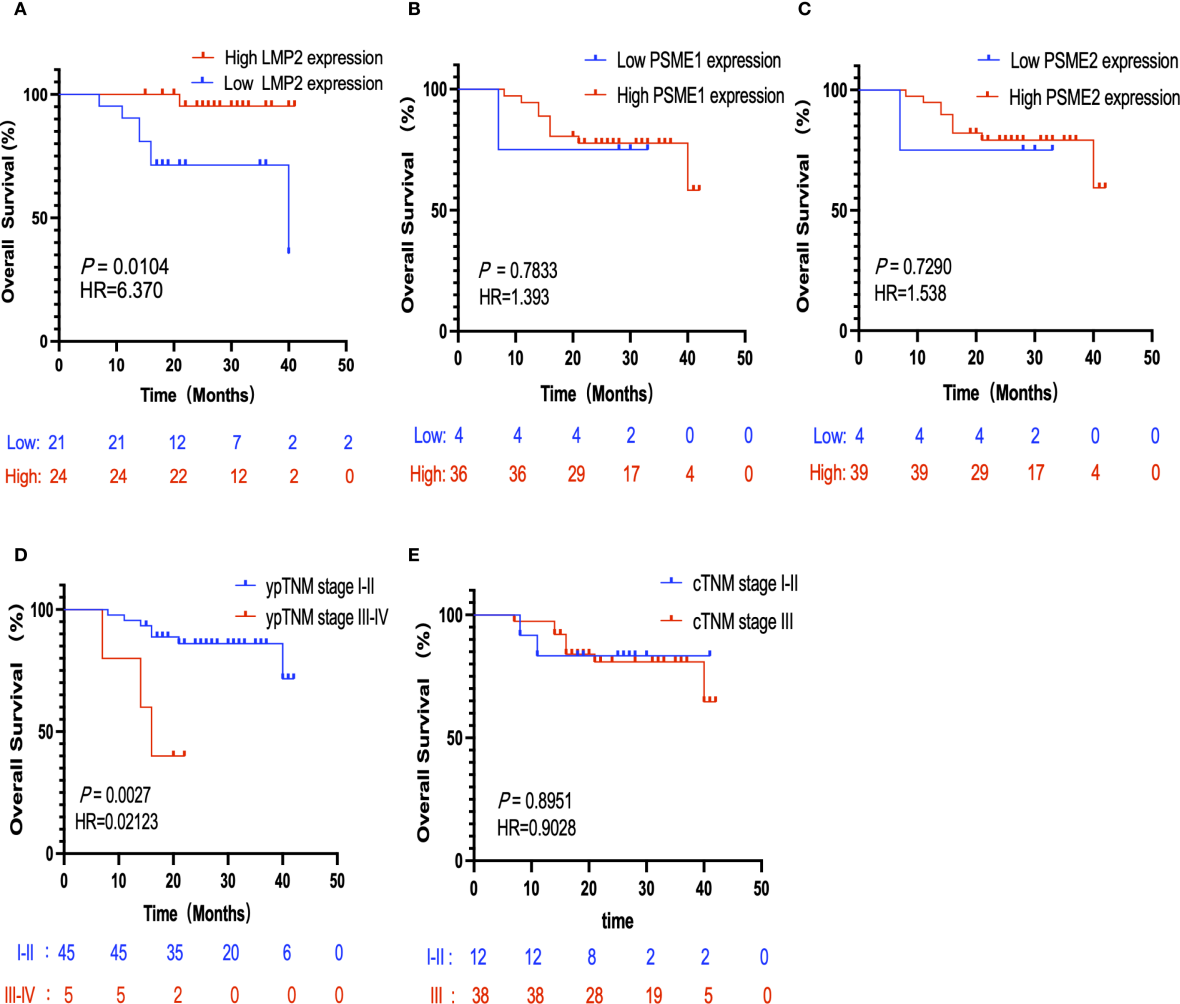
Kaplan-Meier survival analysis. **(A)** Correlation between the expression level of LMP2 in tumor tissues and OS (n=40,*P* = 0.0104). **(B)** Correlation between the expression level of PSME1 in tumor tissues and OS (n=40,*P* = 0.7833). **(C)** The correlation between the expression level of PSME2 in tumor tissues and OS (n=43,*P* = 0.7920). **(D)** The correlation between ypTNM staging and OS (n=50,*P* = 0.0027). **(E)** The correlation between cTNM staging and OS (n=50,*P* = 0.8951).

The threshold for the LMP2, PSME1, PSME2 H-Score is determined by the OS-based X-tile. These patients were divided into low or high groups based on the cut-off value. In order to better explore the prognostic value of LMP2, PSME1 and PSME2 in patients with NACI-NSCLC, Kaplan-Meier survival analysis was used to analyze the changes of LMP2, PSME1 and PSME2 levels. Based on this result, we found that patients with high pre-treatment LMP2 had a significant survival benefit compared to patients with low pre-treatment LMP2 (*P* < 0.0104, median OS: NR vs. 40.0 months) (**Figure 6A**). However, when focusing on PSME1 and PSME2, we found no difference in prognosis between the low and high expression groups of PSME1 and PSME2 in all patients (**Figures 6B, C**). Similarly, we analyzed cTNM and ypTNM to assess their prognostic value in patients with NACI-NSCLC. As expected, we found that ypTNM stage III-IV patients had a worse OS rate than ypTNM stage I-II patients (*P* = 0.0027, median OS: 16.0 months vs. NR), but this phenomenon was not observed in cTNM. In this section, we find that high pre-treatment LMP2 and ypTNM are associated with a favorable prognosis in NACI-NSCLC.

### 3.5 Pre-treatment LMP2 and MPR are independent prognostic factors for NACI-NSCLC

CTNM, ypTNM, pathological response, Histopathology, sex, and pre-treatment LMP2, PSME1, and PSME2 scores were included in univariate and multivariate Cox analyses. Consistent with the above results, univariate Cox analysis showed that MPR, and LMP2 were prognostic factors for NACI-NSCLC OS (**Table 2**). In multivariate Cox analysis, we found that the pre-treatment LMP2 (*P* = 0.007, HR = 0.051,95% confidence interval [CI]: 0.006-0.442) and MPR (*P* = 0.003, HR = 8.709,95% CI: 2.115-35.856) were independent prognostic factors for NACI-NSCLC (**Table 2**).

**Table 2:** Univariate and multivariate Cox analyses of the prognostic factors of overall survival.

Factor	Univariate Cox analysis		Multivariate Cox analysis	
	HR(95%CI)	*P* -value	HR(95%CI)	*P* -value
cTNM stage				
I-II				
III		0.737		
ypTNM stage				
≤II				
III-IV	0.158(0.039-0.639)	0.01	11.316(0.971-131.852)	0.032
Pathology response				
IPR				
MPR	3.833(1.027-14.309)	0.046	8.709(2.115-35.856)	0.003
Histopathology				
Adenocarcinoma				
Squamouss				
Adenosquamous carcinoma		0.427		
Gender				
Male				
Famale		0.186		
LMP2 H-score				
Low				
High	0.107(0.013-0.847)	0.034	0.051(0.006-0.442)	0.007
PSME1 H-score				
Low				
High		0.834		
PSME2 H-score				
Low				
High		0.356		

## 4 Discussion

In recent years, with the rise of immunotherapy, a number of clinical trials are combining neoadjuvant chemotherapy with immunotherapy to achieve better clinical benefits. Chemotherapy drugs can induce immunogenic cell death, which can not only promote the release of tumor antigens and related pro-immunogenic factors into the tumor microenvironment, but also stimulate the uptake of tumor antigens by antigen-presenting cells more effectively, and induce the tumor-specific homologous immune response of cytotoxic T lymphocytes, thereby killing tumor cells (21). These series of effects make neoadjuvant chemotherapy and immunotherapy complement each other (21).

NACI for NSCLC has improved pathological responses and survival rates compared with chemotherapy alone. However, there is still a subset of tumors that do not fully respond to treatment, which is associated with early disease progression. So far, it is impossible to predict these events due to lack of knowledge (11).

So for resectable NSCLC, the identification of effective biomarkers that can predict neoadjuvant treatment efficacy and patient prognosis would undoubtedly help optimize treatment strategies and improve long-term prognosis (22).

Tissue sample-based biomarkers including PD-L1 expression (22), tumor mutation burden (23), and microsatellite instability (24) have been used in clinical practice. However, these biomarkers have several drawbacks, such as high cost, invasiveness, and high heterogeneity of clinical utility owing to differences in the immune status of patients (25). Recently, a phase 2 NADIM trial reported that ctDNA levels correlated significantly with OS in patients with operable NSCLC treated with neoadjuvant nivolumab plus chemotherapy and were superior to radiological assessments in predicting survival However, ctDNA testing is expensive and still at the exploratory stage. There is an urgent need to identify easy-to-use, reliable, and inexpensive biomarkers for identifying patients with NSCLC who may respond to NACI (26).

In this study, we examined the expression of LMP2, PSME1, and PSME2 in pre-treatment NACI-NSCLC tumor tissue specimens and explored their prognostic value in patients with NACI-NSCLC.

We found that low intratumor LMP2, PSME1 and PSME2 expression predicted a good response to NACI in cancer patients. By performing IHC on the biopsy tissues of 50 NSCLC patients receiving NACI treatment, and calculating the corresponding IHC score (cell staining intensity score x positive cell percentage score), we found that those with a low IHC score were more likely to achieve MPR, and vice versa. Those with high IHC score were mostly IPR. Our results suggest that high expression levels of LMP2, PSME1 and PSME2 are associated with positive prognosis in NSCLC patients, and the sensitivity of NSCLC patients to NACI treatment can be predicted in advance by protein expression intensity, thus helping clinicians treat patients more effectively.

In this study, we divided 50 patients with NSCLC into a low LMP2 expression group and a high LMP2 expression group, and performed Kaplan-Meier survival analysis. We found that patients in the high pre-treatment LMP2 group had a significant survival benefit compared with the low pre-treatment LMP2 group (*P* < 0.0104). However, LMP2 expression was low in MPR patients.

Furthermore, to ensure reproducibility and potential clinical translation, we standardized the IHC detection of LMP2, PSME1, and PSME2. Pretreatment core biopsy specimens were processed by routine pathology workflows, including EDTA antigen retrieval, antibody-specific staining, and HRP detection. The H-score (intensity × percentage) was used for scoring, which was independently assessed by two pathologists (mean of 10 HPF) and arbitrated by a third. The cutoff value is determined by X-tile based on the results, which has detection specificity. Before clinical application, the laboratory should lock the threshold through pre-experiment, verify the interobserver repeatability (such as ICC/κ) and maintain batch control.

Compared with the existing technologies such as NGS, PCR and PD-L1 companion diagnosis, triple IHC has shorter reporting cycle and lower incremental cost, which is conducive to promotion in institutions with different resource levels.

As for the integration of diagnostic algorithms, we suggest that it be incorporated into existing diagnostic algorithms for resectable NSCLC, such as performing PD-L1 testing simultaneously or immediately on the initial biopsy specimen. The results can provide a reference for the multidisciplinary team (MDT) to discuss NACI and help identify patients who are likely to benefit or have innate resistance.

In the future, multi-center prospective analysis is needed to evaluate the test consistency and clinical benefit. In addition, the development of more easy-to-implement diagnostic algorithms and algorithms is needed to promote their wider clinical application.

Interestingly, our study revealed a seemingly paradoxical association regarding LMP2 expression: low pre-treatment LMP2 expression predicted a favorable major pathological response (MPR) to neoadjuvant chemoimmunotherapy (NACI) in NSCLC, whereas high LMP2 expression was associated with improved overall survival (OS). This underscores the multifaceted role of LMP2 within the tumor immune microenvironment (TIME). While MPR reflects short-term cytotoxic tumor clearance, OS integrates long-term factors such as immune memory and relapse risk.

We propose the following hypothesis to explain this discrepancy: on one hand, LMP2 may enhance antigen presentation pathways that facilitate the recruitment and activation of cytotoxic T lymphocytes (CD8^+^ T cells), thereby conferring sensitivity to sustained immune activation therapies (e.g., prolonged immune checkpoint inhibitor treatment) and resulting in improved OS. On the other hand, LMP2 may concurrently promote the expansion of immunosuppressive populations such as regulatory T cells (Tregs). During the early treatment phase, these cells can establish an immunosuppressive barrier through the secretion of inhibitory cytokines or contact-dependent mechanisms, thereby reducing the likelihood of achieving an early objective pathological response.

Supporting this notion, our supplementary analysis of the TCGA-LUAD cohort using the CAMOIP platform (http://camoip.net/) demonstrated that high LMP2 expression was associated with increased infiltration of CD8^+^ and CD4^+^ T cells (which may drive long-term antitumor memory and OS benefit), but also with higher proportions of Tregs (**Supplementary Figure S1**), potentially establishing early immune tolerance and impairing MPR.

In addition, LMP2 is a critical catalytic subunit of the immunoproteasome, which has been widely reported to more efficiently process proteins into antigenic peptides for presentation by MHC-I molecules (27, 28). Compared with the standard proteasome, the immunoproteasome generates peptides with higher binding affinity to MHC-I, thereby potentially enhancing T-cell recognition (29). Consistent with this, our exploratory analysis via CAMOIP (http://camoip.net/) further showed that tumors with high LMP2 expression exhibited higher expression levels of MHC molecules (HLA genes), which aligns with previous findings in the literature (**Supplementary Figure S2**).

In recent years, controversy remains as to whether PCR and MPR represent surrogate end points for EFS and OS in neoadjuvant trials for resectable NSCLC. Hines JB et al. (30) searched archives of PubMed and international conference abstracts from June 2017 to October 31, 2023. Studies incorporating a neoadjuvant arm with immune checkpoint blockade alone or in combination with chemotherapy were included. For trial-level surrogacy, log ORs for PCR and MPR and log hazard ratios for EFS and OS were analyzed using a linear regression model weighted by sample size. The regression coefficient and R^2^ with 95% confidence interval were calculated by the bootstrapping approach. A strong correlation was revealed between PCR and MPR and 2-year EFS, but not OS.

In summary, we preliminarily conclude that patients in the high pre-treatment LMP2 group have a better survival benefit (OS length), that low pre-treatment LMP2 expression is more likely to achieve MPR, and that while MPR is undoubtedly a potential endpoint of exciting clinical benefit, it is not yet a sufficient proxy for OS in clinical trials. Given that PCR, MPR and EFS are closely related at the patient level, these endpoints provide valuable data for clinical treatment decisions. Currently, these endpoints should be considered co-primary endpoints in clinical trials evaluating the benefits of neoadjuvant and perioperative immunotherapy. This may change as neoadjuvant and perioperative immune checkpoint blocking studies mature.

However, when we looked at PSME1 and PSME2, we found no difference between the groups with high or low expression of PSME1 and PSME2. In addition, we concluded that ypTNM stage I-II patients had a better survival benefit (*P* = 0.0027). However, this phenomenon has not been observed in cTNM. Therefore, we conclude that MPR, high LMP2, and ypTNM are favorable prognostic factors for patients with surgically resectable NACI-NSCLC.

In multivariate Cox analysis, we found that LMP2 and MPR before neoadjuvant therapy were independent prognostic factors for OS. This result is consistent with Kaplan-Meier survival analysis.

Whereas, in this study, some limitations cannot be ignored. First, this was a single-institution retrospective study, so there may be selection bias. Second, many patients are excluded because pre-treatment protein levels cannot be assessed. Only 50 patients with NACI-NSCLC were included in this study, a small number of whom were unable to obtain a pre-treatment biopsy specimen. Third, the number of patients included in this study was relatively small and the follow-up time was short. Fourth, a key limitation is the absence of functional validation. While our study established associations between LMP2, PSME1, PSME2 expression (via transcriptomic/database analysis and IHC in a clinical cohort) and NACI response/survival outcomes, the IHC correlations do not establish a causal role for these immunoproteasome subunits in anti-tumor immunity. To address this, future studies will prioritize systematic functional investigations, including: knockdown/overexpression in NSCLC cell lines; flow cytometry and T-cell co-culture assays for activation/cytotoxicity; evaluation of therapeutic sensitivity in immunocompetent syngeneic mouse models; and multiplex IHC/IF on existing FFPE samples to delineate spatial protein-immune infiltration relationships. These experiments are crucial for validating and extending the current findings. Finally, in general, disease-free survival is the primary endpoint of NACI-NSCLC studies, but in this retrospective study, we explored the prognostic value of the proteasome complex, which in part reflects antitumor immunity. In addition, anti-tumor immunity is considered to be an important prognostic factor for OS in NSCLC patients, so we selected OS as the study endpoint.

In summary, our study showed that in resectable NSCLC, patients with low intratumor expression of LMP2, PSME1 and PSME2 had a better response to NACI than those with high expression. Meanwhile, we found that LMP2 and MPR before neoadjuvant therapy were independent prognostic factors for patients with NACI-NSCLC. Finally, with a deeper understanding of the molecular mechanisms underlying the formation, function, and therapeutic benefits of LMP2, PSME1, and PSME2 in the tumor, it may help guide NACI’s strategies for personalized precision medicine in the future.

## 5 Conclusion

This study confirms that the expression levels of LMP2, PSME1, and PSME2 can serve as potential biomarkers for the efficacy and prognosis of NACI in patients with NSCLC. The results show that the low expression of LMP2, PSME1, and PSME2 within the tumor is significantly correlated with a good pathological response (MPR) of patients to NACI, suggesting that they may influence treatment sensitivity by regulating antigen presentation and the immune microenvironment. In addition, high initial LMP2 expression and MPR status have been identified as independent prognostic factors for the OS of patients, providing a new basis for clinical stratified treatment. However, this study has the limitations of a single-center retrospective design, a relatively small sample size, and a short follow-up period, which may affect the generalizability of the results. In the future, multi-center, prospective cohort studies are needed to further validate the clinical application value of these biomarkers. Moreover, it is necessary to explore their potential role in optimizing individualized NACI treatment in combination with molecular mechanism research, so as to improve the long-term survival benefits of patients.

Recent phase II/III clinical trials have confirmed that NACI is an effective treatment strategy for resectable NSCLC (4–9), but response heterogeneity remains a major clinical challenge. Current tissue- and blood-based biomarkers (PD-L1, TMB, ctDNA, TILs) provide partial predictive value but have practical limitations, including cost, invasiveness, and variability in detection (22, 31–34). Our study proposes immunoproteasome components (LMP2, PSME1, PSME2) as candidate tissue biomarkers measurable by routine IHC, which may complement existing approaches. For future work, we suggest: first, prospective validation in multi-center cohorts is essential; second, integrating LMP2/PSME1/PSME2 with established biomarkers (such as PD-L1) could improve predictive accuracy; and third, mechanistic studies should explore how alterations in the immunoproteasome affect antigen processing and T-cell recognition in the NACI context. Specifically, subsequent research should aim to: (1) standardize IHC scoring of LMP2/PSME1/PSME2 across laboratories using digital pathology platforms; (2) incorporate these biomarkers into ongoing neoadjuvant trials to validate their clinical utility; and (3) establish standardized scoring and reporting guidelines to accelerate clinical translation.

## Data Availability

The raw data supporting the conclusions of this article will be made available by the authors, without undue reservation.
