# Supplementary material for: Immunoproteasome components LMP2, PSME1, and PSME2 as novel tissue biomarkers predicting response and survival in neoadjuvant chemoimmunotherapy for resectable NSCLC
Source: Front Immunol. 2025 Sep 16;16:1654573. doi: 10.3389/fimmu.2025.1654573 (PMC12479417; doi:10.3389/fimmu.2025.1654573)
Supplement: Supplementary file 1 [file DataSheet1.docx]

Supplementary Material

# Supplementary Figures and Tables

## Supplementary Figures


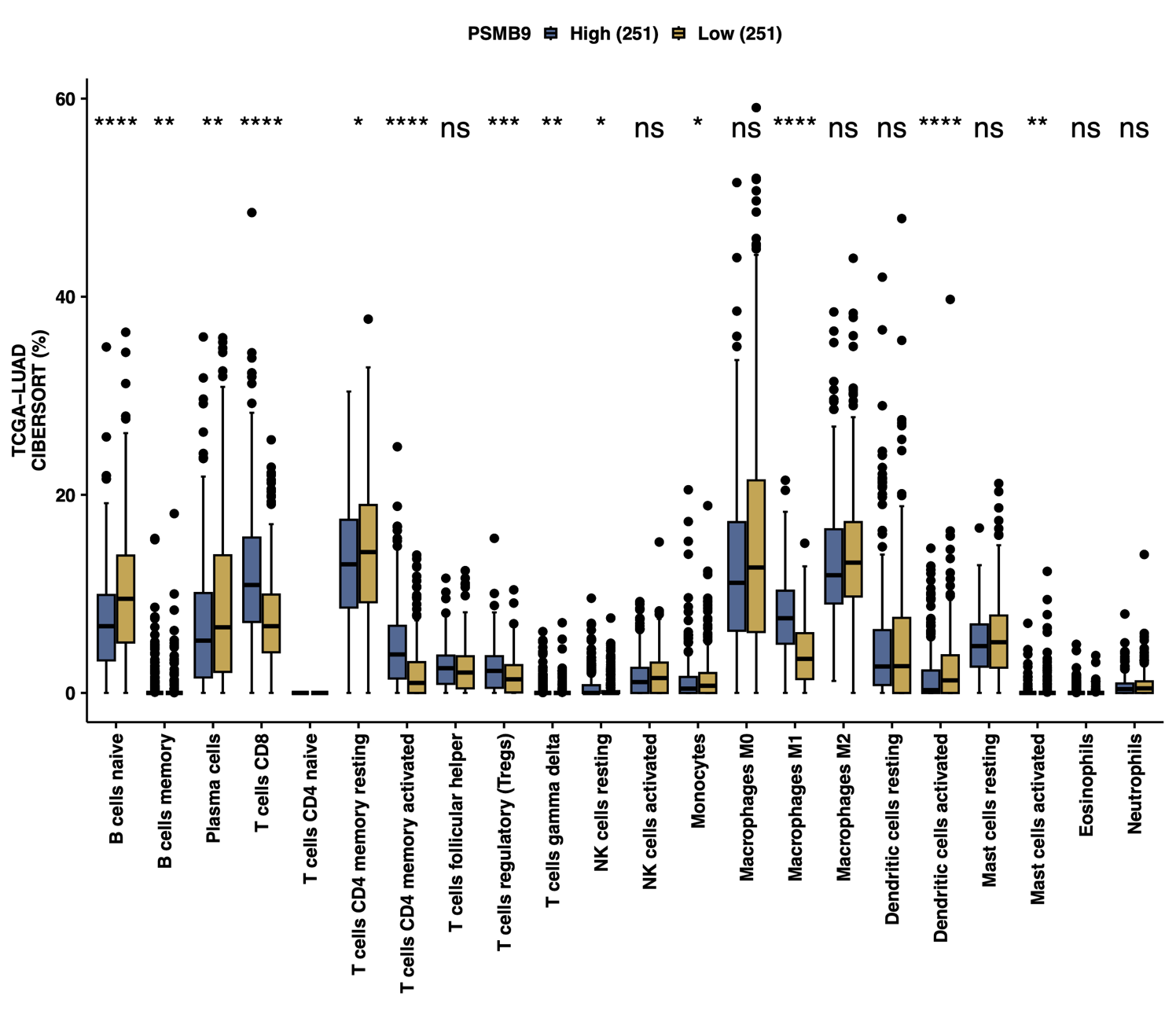


**Supplementary Figure 1.** **Immune cell infiltration analysis in TCGA-LUAD cohort stratified by PSMB9 (LMP2) expression.**

The figure illustrates the proportions of 22 immune cell types estimated by CIBERSORT in patients with high (n=251) versus low (n=251) PSMB9 expression. High PSMB9 expression is associated with increased infiltration of CD8⁺ T cells and CD4⁺ T cells, which may contribute to antitumor immunity and long-term survival. However, an elevated proportion of regulatory T cells (Tregs) is also observed, which may promote immune tolerance and potentially impair major pathological response (MPR). Analysis was performed using the CAMOIP platform (<http://camoip.net/>).

**
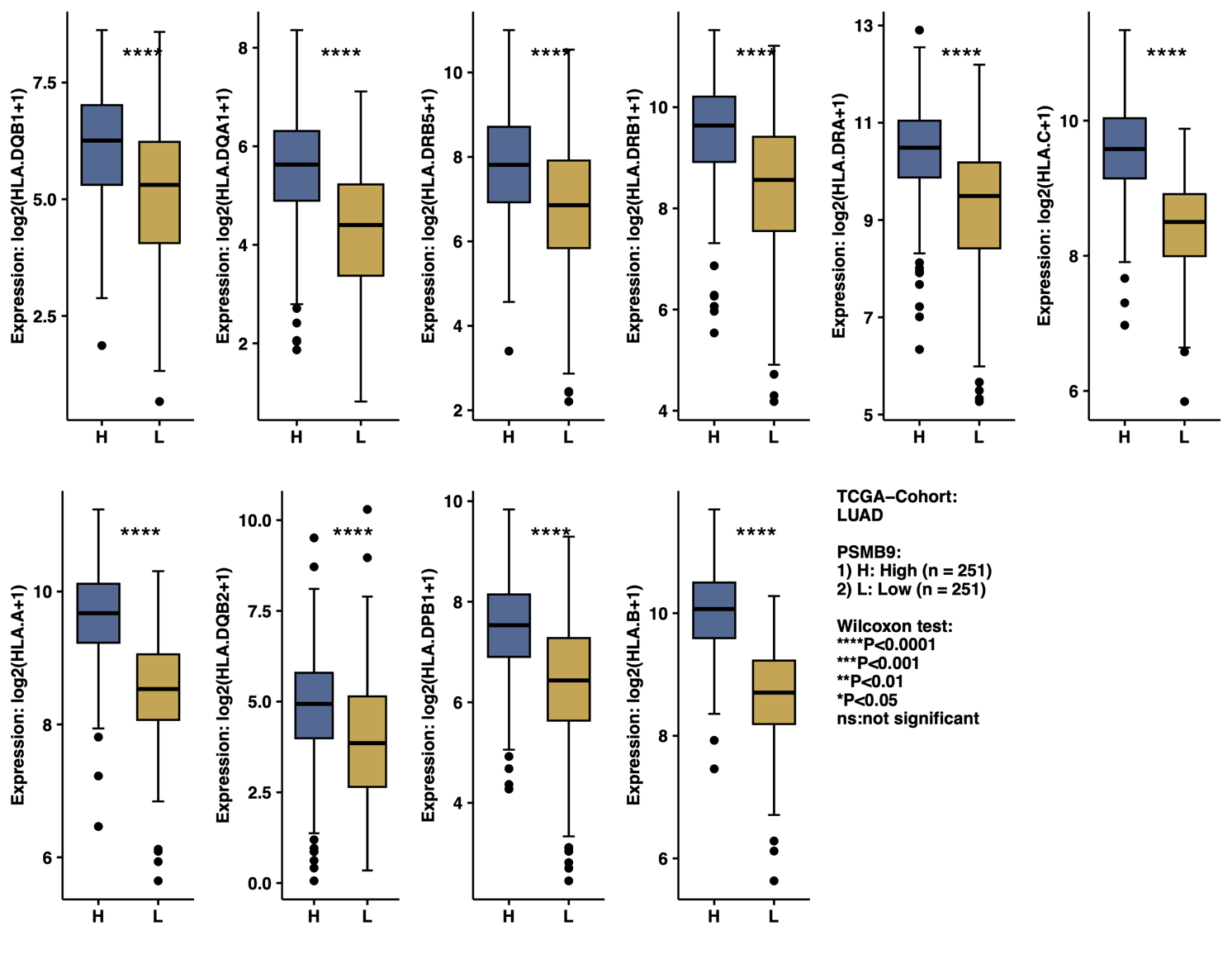
**

**Supplementary Figure 2. Expression levels of MHC class II HLA genes in tumors with high versus low PSMB9 (LMP2) expression.**The boxplots show log2-transformed expression values (log2(TPM+1)) of multiple HLA gene variants in the TCGA-LUAD cohort. Tumors with high PSMB9 expression exhibit upregulated expression of HLA genes, consistent with enhanced antigen presentation capability via MHC-II molecules. This aligns with the role of LMP2 as a catalytic subunit of the immunoproteasome, which

facilitates efficient peptide processing and presentation. Analysis was conducted using the CAMOIP platform (<http://camoip.net/>).

1. **Supplementary Tables**

**Table.S1 Quantification of gene screening**

| **Study** | **Response_Group** | **Gene** | **title** |
| --- | --- | --- | --- |
| Study1 | Reaction-related_Top | *CD137,* ***LMP2(PSMB9)*** | Immune gene signatures for predicting durable clinical benefit of anti-PD-1 immunotherapy in patients with non-small cell lung cancer |
| Study2 | Reaction-related_Top | ***PSME1,*** ***PSME2 , LMP2(PSMB9)*** *,VGF and CD8+ T* | Genomic and transcriptomic analysis of checkpoint blockade response in advanced non-small cell lung cancer |
| Study3 | Reaction-related_Top | *cytotoxic cells, NK cells, Th1 cells, cluster 2, CD8 T cells, cluster 1, T cells, cluster 4, cluster 3, CD45, CD4-activated, dendritic cells, neutrophils and Treg cells* | Immune-Related Gene Expression Profiling After PD-1 Blockade in Non–Small Cell Lung Carcinoma, Head and Neck Squamous Cell Carcinoma, and Melanoma |
| Study4 | Reaction-related_Top | *APM (antigen processing machinery) : B2M, TAP1, TAPBP,* ***PSME1, PSME2,*** *CALR,* ***LMP2(PSMB9), PSMB10,*** *ERAP1, PDIA3, NLRC5, RFX5, PSME3, CIITA, HSP90AB1, HSP90AA1 and HSP90B1* | Gene signature of antigen processing and presentation machinery predicts response to checkpoint blockade in non-small cell lung cancer (NSCLC) and melanoma |
| Study5 | Reaction-related_Top | *ERAP1; ERAP2;* ***LMP2(PSMB9);*** *ERAP1 rs26653; ERAP1 rs30187; ERAP1 rs27044* | Polymorphisms of Antigen-Presenting Machinery Genes in Non-Small Cell Lung Cancer: Different Impact on Disease Risk and Clinical Parameters in Smokers and Never-Smokers |
| Study6 | Reaction-related_Top | *DPYD, GALK1, CDC23, UBE2L3, RHEB and* ***PSME1*** | Liquid biopsy-derived extracellular vesicle protein biomarkers for diagnosis and prognostic assessment of lung squamous cell carcinoma |
| Study7 | Reaction-related_Top | ***LMP2(PSMB9),*** *MECL-1 and LMP7* | Pro- and anti-tumorigenic capacity of immunoproteasomes in shaping the tumor microenvironment |
| Study8 | Reaction-related_Top | *PSMB8,****LMP2(PSMB9), PSMB10, PSME1, PSME2,*** *IRF1* | IP-score correlated to endogenous tumour antigen peptide processing: A candidate clinical response score algorithm of immune checkpoint inhibitors therapy in multiple cohorts |

**Table.S2**  **IHC Scoring Data**

| **Patient** | **LMP2** | **PSME1** | **PSME2** | **MPR-0/IPR-1** |
| --- | --- | --- | --- | --- |
| 1 | 4 | 1 | 3 | 0 |
| 2 | 2 | 6 | 4 | 1 |
| 3 | 4 | 8 | 12 | 1 |
| 4 | 4 | 6 | 3 | 0 |
| 5 | 6 | 8 | 8 | 0 |
| 6 | 3 |  |  | 1 |
| 7 | 6 | 6 | 6 | 0 |
| 8 | 12 | 8 | 12 | 1 |
| 9 | 8 | 4 | 8 | 0 |
| 10 | 3 | 6 | 6 | 0 |
| 11 | 12 |  | 9 | 1 |
| 12 | 2 | 3 | 2 | 0 |
| 13 | 6 | 6 | 6 | 0 |
| 14 | 2 | 3 | 6 | 0 |
| 15 | 1 | 1 | 2 | 0 |
| 16 | 6 | 3 | 8 | 0 |
| 17 | 3 | 3 | 3 | 0 |
| 18 | 12 | 8 | 12 | 1 |
| 19 | 3 | 2 | 8 | 0 |
| 20 |  |  | 2 | 0 |
| 21 | 8 |  | 6 | 0 |
| 22 |  | 3 |  | 0 |
| 23 |  | 8 | 12 | 1 |
| 24 | 6 |  |  | 0 |
| 25 | 6 | 4 | 8 | 1 |
| 26 | 1 | 2 | 3 | 0 |
| 27 | 4 |  |  | 0 |
| 28 | 2 |  |  | 0 |
| 29 |  |  |  | 0 |
| 30 |  | 2 | 3 | 0 |
| 31 | 6 | 2 | 6 | 0 |
| 32 | 12 | 3 | 8 | 0 |
| 33 | 12 | 8 | 12 | 1 |
| 34 | 6 | 4 | 12 | 1 |
| 35 | 4 | 6 | 6 | 0 |
| 36 | 2 | 4 | 9 | 1 |
| 37 | 3 | 4 | 8 | 0 |
| 38 | 12 | 12 | 12 | 1 |
| 39 | 2 | 1 | 3 | 0 |
| 40 | 2 | 8 | 12 | 1 |
| 41 | 2 |  | 4 | 0 |
| 42 | 1 | 3 | 3 | 0 |
| 43 | 3 |  | 6 | 0 |
| 44 | 1 | 4 | 3 | 0 |
| 45 | 2 | 6 | 6 | 0 |
| 46 | 6 | 6 | 4 | 0 |
| 47 | 3 | 6 | 6 | 0 |
| 48 | 9 | 12 | 12 | 1 |
| 49 | 4 | 8 |  | 0 |
| 50 | 2 | 1 | 2 | 0 |
