# Supplementary figures and images for: Immunoproteasome components LMP2, PSME1, and PSME2 as novel tissue biomarkers predicting response and survival in neoadjuvant chemoimmunotherapy for resectable NSCLC
Source: Front Immunol. 2025 Sep 16;16:1654573. doi: 10.3389/fimmu.2025.1654573 (PMC12479417; doi:10.3389/fimmu.2025.1654573)

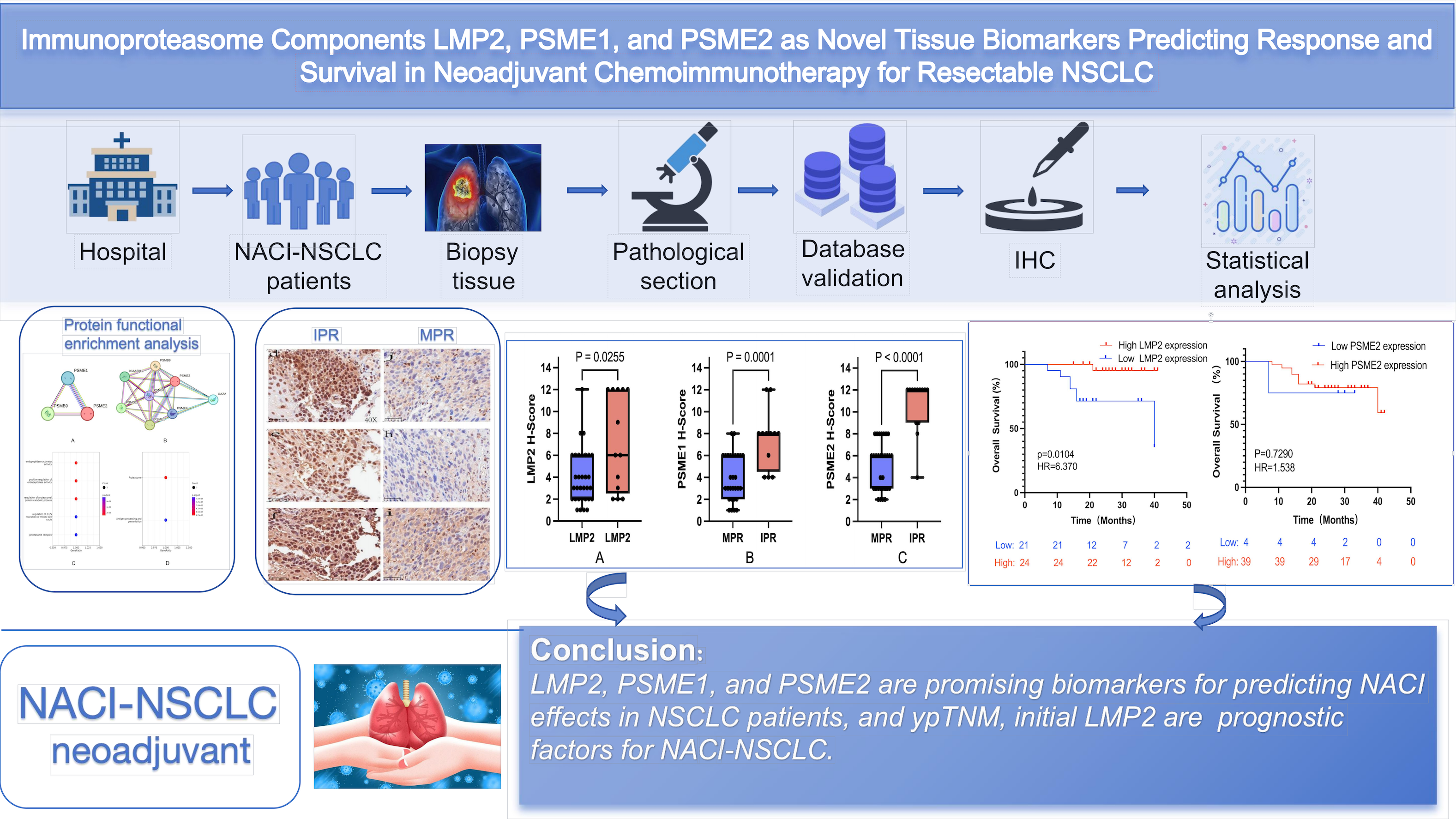

Supplement: Supplementary file 2 [file Image1.tif]
